# Strengthening primary health care: contributions of young professional-led communities of practice

**DOI:** 10.1017/S1463423621000815

**Published:** 2022-03-02

**Authors:** Kaara Ray B. Calma, Lynsey J. Brown, G.V.M. Chamath Fernando, Lundi-Anne Omam

**Affiliations:** 1 University of Wollongong, School of Nursing, Faculty of Science, Medicine and Health, Wollongong NSW, Australia; 2 Research Fellow, Flinders University of South Australia, Adelaide, SA, Australia; 3 Division of Health Research, Faculty of Health and Medicine, Lancaster University, Lancaster, UK; 4 National Centre for Primary Care and Allergy Research, Department of Family Medicine, Faculty of Medical Sciences, University of Sri Jayewardenepura, Nugegoda, Sri Lanka; 5 Department of Public Health and Primary Care, University of Cambridge, Cambridge, UK

**Keywords:** capacity building, collaboration, community of practice, global health, leadership, primary health care, youth, youth engagement

## Abstract

**Background:**

Health systems that have strong primary health care at their core have overall better patient outcomes. Primary health care is key to achieving Universal Health Coverage and the broader health-related Sustainable Development Goals by 2030. In 2018, at the launch of the Declaration of Astana, the World Health Organization formed the inaugural Primary Health Care Young Leaders’ Network.

**Objective:**

This paper aims to demonstrate the scope for young professional-led communities of practice in fostering support systems for young leaders and strengthening the delivery of primary health care at multiple levels.

**Methods:**

A description of the Young Leaders' Network community of practice model is presented, with examples of the work the members are doing, individually and collectively, to advance the science and practice of primary health care.

**Results:**

This initiative brought together 21 individuals from across the world, working across disciplines and within an array of socioeconomic contexts to improve primary health care in their respective countries.

**Conclusions:**

This youth-led community of practice is able to share knowledge, evidence and resources to inform clinical and public health activities, policy initiatives, advocacy and research to improve primary health care delivery and health outcomes for communities across the globe.

## Youth engagement in health

As the global population surpassed 7 billion in 2012, a little over 50% of it constituted those aged 30 years or younger (Johnston *et al.*, 2019). The role of young people as key agents for social change, economic growth and technological innovation has long been recognised by the United Nations (UN) (Bulc *et al.*, 2019; Marcus & Cunningham, 2016; United Nations, 2013). Evidence has shown that as educators, young people have the power to challenge negative attitudes in a manner that is both impactful and engaging, which makes them well positioned to drive change and influence their communities (Marshall, 2019; Struthers *et al*., 2019). The transformational potential of young people to drive progress towards universal health coverage can, however, only be achieved through participatory leadership (Bulc *et al*., 2019; Marcus & Cunningham, 2016). As such, youth engagement has never been more vital to ensure actions meet these generations’ needs and to help young people reach their potential.

Partnerships between young people and more senior experts to drive meaningful change have been led and facilitated by various organisations, including intergovernmental organisations, donors and foundations, agencies of the UN, and the World Health Organization (WHO) (Checkoway, 2011; UN, 2013; Zeldin *et al*., 2013). The UN has acknowledged the need to work for and with young people in achieving the Sustainable Development Goals (SDGs). With the launch of the UN Youth Strategy (Office of the Secretary-General’s Envoy on Youth, 2018) in 2018, the WHO committed to action on meaningful youth engagement. The WHO released a global report citing strategic recommendations, with emphasis on: (1) leadership; (2) country impact; (3) focusing global public goods on impact and (4) partnerships (WHO, 2018b). The review identified more than 60 organisations that are potential partners with WHO in young people’s engagement on health. However, funding to support young people’s enthusiasm and make space for this demographic remains a challenge. Knowing the challenges ahead, the report acknowledged the need to forge creative new partnerships with diverse organisations that engage with young people and to work with partners to identify and develop potentially innovative funding sources (WHO, 2018b). In the primary health care (PHC) space, youth-led organisations such as the International Federation of Medical Students’ Associations and International Pharmaceutical Students’ Federation maintain official partnerships with the WHO. Despite their indispensable contributions, youth voices in the global health policy-making arena have been limited (Lal *et al.*, 2019). Groups such as the Young Primary Care Experts of the European Forum for Primary Care (You&EFPC), the Young Forum Gastein of the European Health Forum Gastein (YFG-EHFG) and the Youth Forum of the European Public Health Association (EUPHANxt) have passionately represented the youth spirit on the common public health agenda in the European region over a decade. Nevertheless, a youth movement with a global membership with diverse traits, skillsets and experiences that is able to create a collective momentum towards achieving goals of global interest neither existed nor was publicly envisioned by any public health organisation.

In light of this, the WHO launched the PHC Young Leaders’ Network (YLN; WHO, 2018c) at the Global Conference on PHC in Astana in 2018. The convening of young professionals as a community of practice (CoP) emphasised the key role of youth in improving PHC and delivering on the Astana Declaration (WHO, 2018a). CoPs typically comprise of mutual engagement, joint enterprise, shared repertoire, collective competence, shared identity, commitment to a cause, collective learning, thinking together and regular interactions (Li *et al.*, 2009; Pyrko *et al*., 2017; Wenger, 2011). The attributes, dynamics and activities of the YLN described in this paper portray how they comprise a CoP. This paper demonstrates the scope for young professional-led CoPs in fostering support systems for young leaders and strengthening the delivery of PHC at local, regional and global levels; with examples from the YLN.

## The YLN CoP

The YLN’s mission encompasses working individually and through interdisciplinary collaboration to advance the science and practice of PHC. This CoP was formed with two main ambitions: (1) to drive authentic engagement with youth in PHC and (2) to leverage meaningful engagement to improve PHC in the communities members work in. The 21 members were selected from over 2000 applicants as those who had demonstrated outstanding leadership qualities in PHC development in their respective regions. The YLN’s strength is the membership’s diverse expertise. Akin to PHC’s broad scope, the WHO recognised the importance of recruiting professionals from a variety of disciplines and socio-economic contexts across all six WHO regions (see Table 1).

**Table 1:** Composition of the inaugural Young Leaders’ Network

WHO region	Country	Area of expertise
Africa	Cameroon	Civil society and public health expert involved with developing and managing health interventions in hard-to-reach, post-conflict and conflict-affected communities
	Ghana	District public health nurse and district Community-Based Health Planning and Services coordinator
	Malawi	Registered nurse and midwife, maternal and child health lead nurse
	Nigeria	General practitioner and public health expert, managing Result-Based Financing in the northeast region of Nigeria through the World Bank assistance. Postgraduate Fellow of public health providing humanitarian support to the displaced population through international aids
The Americas	Chile	PhD candidate of public health, social worker and technical advisor for the Ministry of Health
	United States of America (California)	Sociologist and public health rights advocate among the underserved populations
	United States of America (Alaska)	Academic in social medicine and global health advocating for health rights among rural communities
South East Asia	Indonesia	Primary care physician with leadership and managerial experience, advancing quality assurance in primary care through the National Cost and Quality Control Team Committee
	Sri Lanka	General practitioner, collaborative researcher and academic in a leading medical school, with a special interest in palliative care medicine
Europe	Italy	Family physician currently employed with Médecins Sans Frontières, and an executive board member of the European Forum for Primary Care
	Kazakhstan	General practitioner and family medicine trainer with a particular interest in domestic violence and quality improvement in primary care
	Portugal	General practitioner with a special interest in palliative care, and President for the Global Young Family Doctors’ movement
	United Kingdom	General practitioner, member of the Royal College of General Practitioners’ Junior International Committee also supporting local and national HIV and sexual health programmes
Eastern Mediterranean	Pakistan	Epidemiologist involved in infectious diseases research, monitoring and evaluation and community-led health promotion initiatives at a national level
	Pakistan	Public health programme manager dedicated to reducing neonatal and child mortality in peri-urban communities through longitudinal coordinated PHC provision
	United Arab Emirates	Public health activist, health educator and member of the healthy lifestyle policy team of the Ministry of Health, Dubai
Western Pacific	Australia	Research Fellow with a background in health psychology and public health, involved in PHC knowledge exchange, health promotion and practice-based research into quality improvement in general practice
	Australia	Registered nurse, lecturer and PhD candidate whose interests include PHC nursing, undergraduate nursing curriculum development and health experience
	Philippines	Registered nurse and public health professional working in the field of PHC, sexual reproductive health and rights including HIV. Also has experience in advancing and upholding the rights of young people, including women
	Philippines	Medical doctor dedicated to remote health care provision currently affiliated with the Social Innovation in Health Initiative Philippines Hub
	Vietnam	Primary care physician and Internist, Director of the Program in Global Primary Care and Social Change at Harvard Medical School, and Associate Director in health systems strengthening for the Partnership for Health Advancement in Vietnam

The YLN provides a unique PHC voice among those advocating for youth engagement (*Bulc et al.*, 2019), but beyond that, it has an impact through members making changes in their own settings. It is important that youth movements such as this demonstrate not only what youth can do but what youth are already doing. Box 1 illustrates some of the successful strategies the YLN CoP adopted to produce positive outcomes during their term and as they continue engagement. These facilitators can be used to inform the development of similar networks in the future.

Box 1. Key elements in building a CoP to strengthen PHC

Facilitation (bringing together PHC professionals from around the world)

- Influential organisational lead as initial coordinator
- Group comprised of multidisciplinary, cross-country professionals
- Opportunities to engage with senior experts/access to mentors
- Provision of project opportunities (e.g., presentations, input to technical documents) and support for CoP-led projects (e.g., collaborative research)
- Lead coordinator committed to listening openly to ideas presented by members

Activities (driving change to strengthen PHC)

- Shared passion for PHC, vision and goals of achieving health for all
- Developing professional identity
- Cross-disciplinary learning underpinned by mutual respect and openness
- Formal and informal methods for providing clinical and research advice to each other
- Requesting feedback on WHO documents (e.g., Operational Framework)
- Publishing regular blogs in WHO’s PHC newsletter, featuring YLN members’ work and perspectives
- Ongoing training and skill development
- Scaling up advocacy
- Connecting with other youth-led agencies

Fit-for-purpose communication tools (exchanging ideas to inform future PHC initiatives)

- Regular opportunities to connect
- Face-to-face meetings facilitating a platform of support and opportunities to develop key connections
- Informal gatherings
- Use of web-based platforms for formal discussion fora
- Use of social media (e.g., WhatsApp) for easy, rapid questions, sharing of resources and provision of support

## The benefits of young professional-led CoPs for PHC

Specific attributes of the YLN manifest in significant impacts within and outside the CoP. For instance, involving young professionals with experience from critical frontlines of PHC practice, research and policy in different regions means a wealth of opportunities for members to learn from each other. These interactions enhance critical thinking, thus leading to developing sustainable solutions for the challenges within members’ own contexts (Withers *et al*., 2016). A CoP not only benefits the members but also the communities to which they belong, by increasing social competence and embedding a sense of social responsibility.

The WHO engaged the network by supporting a number of diverse activities, including webinars, mentorship links and in-person meetings. These engagements have enhanced each members’ leadership skills and in turn have influenced their individual work and output in their respective countries. At a local level, CoPs such as the YLN offer insights into current training processes and frontline experiences of PHC service providers, researchers and policy-makers. They promote access to information about what works in PHC and which elements need strengthening. They also facilitate relationships between individuals; working in partnership with other CoP members allows individuals to build capacity and learn from others enacting similar programmes in different areas. For example, Rehan Adamjee from Pakistan works with a local non-governmental organisation, and the Aga Khan Medical College to deliver longitudinal, coordinated PHC services to communities on the outskirts of Karachi. The work has helped to reduce the under-5 mortality in these areas by 40% over a 4-year period. Rehan has leveraged insights from global professionals through the YLN, and through meeting other practitioners at global health events. His growing experience has helped him refine his work within his local community and expand it to new communities across Karachi. Similarly, Edmund Duodu is a District Public Health Nurse from Ghana who is currently providing crucial PHC services in hard-to-reach communities through his community-based organisation, ‘Divine Mother and Child Foundation’. Through the YLN, he has been able to meet and work with senior health officials in Ghana to drive local improvement strategies towards strengthening PHC.

Another young leader whose work experience in the YLN contributed to improving her work as a PHC professional is Victoria Gichohi. Victoria currently works as a health educator in the Los Angeles County Department of Public Health. In this role, Victoria has provided training to caregivers and health care administrators on the basics of COVID-19, which has been funded by a grant from the Centers for Disease Control and Prevention. Victoria has facilitated virtual training to over 200 participants in Los Angeles County. Through her participation in the YLN, she has gained the proper tools to support and assist this population. To date, Victoria stays passionate in helping underserved communities within Los Angeles, USA.

At a country or regional level, bringing together young professionals from diverse areas enhances knowledge exchange across countries. For example, Dr. Audu Lucky Emmanuel is a general practitioner who has served vulnerable populations in northeast Nigeria through humanitarian support and strengthening government institutions through performance-based financing. He led a team of PHC workers in rural Borno state, providing comprehensive PHC to those affected by the ongoing Boko Haram terrorist conflict. His YLN involvement has enabled him to study primary care in fragile health systems under one of the field’s global experts. Dr. Audu was able to link with global health professionals at the 2019 Primary Care 2030 Dialogue in Dubai. Through connecting with YLN member Dr. David Duong (physician, technical advisor and programme director), Dr. Audu was able to interact with PHC stakeholders from Kenya and also Vietnam, where he gained knowledge about innovative digital strategies being implemented in these regions, and took these lessons back to inform initiatives in his own region.

Certain resourceful collaborations the team members developed through attending the global health events have enabled ongoing benefits to their respective regions, beyond the YLN’s initial term. For instance, Dr. Chamath Fernando, a Sri Lankan academic General Practitioner, and a palliative care enthusiast, through the relationships he had formed with the International Association for Hospice Palliative Care through the years, is currently attracting expertise and guidance from a wealth of global primary palliative care clinicians and academics towards the development of a Postgraduate Diploma in Palliative Care for Sri Lankan General Practitioners. Furthermore, he anticipates working further with the Asia Pacific Primary Palliative Care Special Interest Group that was derived recently consequent to these collaborations, towards attaining regionally shared agendas.

CoPs can have global impact by connecting advocates around the world. For example, the YLN members represent 17 countries. This provides a glimpse into PHC in different contexts and enables the widespread, global dissemination of new information, new technologies and new skills. Technology facilitates regular communication within CoPs, mitigating the restraints imposed by geographical boundaries. For example, the YLN leverages social media such as WhatsApp for problem-solving. These tools are indispensable in allowing members to connect more liberally and rapidly thus mobilising support, advice and resources. For instance, as physician Dr. Andrea Canini sought advice to roll out an immunisation initiative within Sierra Leone, guidance came via WhatsApp from colleagues such as Lundi-Anne Omam Ngo Bibaa who had trialled similar approaches through her humanitarian work in Cameroon.

When needing a ‘deep dive’ into key policy or practice areas, formal platforms can be used for regular webinars and discussion fora. For example, during the COVID-19 crisis, the YLN members partnered with another international PHC programme for a webinar series, sharing insights on frontline experiences of pandemic responses and the future of PHC in the post-pandemic era (Harvard Medical School Center for Primary Care, 2020). This global series was evidence that once established, the YLN has remained viable (independent of WHO facilitation) and adaptable to the most pressing health issues in the world. The value of frontline PHC perspectives was highlighted in these webinars as members presented lessons from their experiences to rapidly problem-solve, share knowledge and adapt successful strategies to their different contexts. This was only possible by having strong connections and communications well established prior to the pandemic.

## What is the future for youth-led PHC CoPs?

The YLN (and similar CoPs) must acknowledge some barriers in terms of sustainability. These include issues such as time commitment among already busy members, tackling time differences in trying to organise meetings, accounting for changes in priorities (e.g., shift away from a focus on SDGs to frontline support for COVID-19) and maintaining commitment without funding to support face-to-face connections or an ongoing facilitator to support regular meetings. However, young professional-led CoPs are underpinned by passion and determination to improve intergenerational futures. These barriers are thus overcome by agility; with activities such as supporting different means of connecting (i.e., social media, web-based communication forums that can be accessed when convenient to members), encouraging champions within CoPs to step up and take on a facilitation role when there is no longer a formal coordinator in place, and changing agendas to reflect current priorities/adapting goals to reflect the wider context. The YLN represents a community that is striving to make a real difference. While meetings are less frequent than in the initial stages, communication and shared activities continue, enabling progress.

Youth-led movements have great potential to inspire other young people, as well as future generations, to make meaningful contributions to their communities. By recruiting members from clinical, policy and academic settings, the YLN’s breadth of experience models young professionals’ diverse skillsets. It should be the goal of every youth network to continue communication with other youth groups and more experienced experts globally. By building connections now, young people can foster a larger worldwide network that is prepared to take on the significant PHC roles and responsibilities that await them.

Whilst there are rapid technological and scientific advancements to aid the delivery of PHC, there remain many challenges. One challenge is the pervasive spread of misinformation on social media platforms, such as with vaccinations (Smith & Graham, 2019). In light of this, young leaders acknowledge their obligation to spread evidence-based information on trusted platforms that are easily accessible to communities. In this way, young PHC professionals remain embedded within the WHO Youth Engagement Strategy, with the WHO engaging young people through the design and delivery of global public goods and in innovative partnership-driven platforms where they can continue sharing experiences whilst driving change (WHO, 2018b).

Through their research, education and training, or direct fieldwork, it is the young leaders’ goal to continue their involvement in addressing PHC workforce shortages by decreasing siloes through the integration of care teams (i.e., allied health, social care, community health workers, civil society and tertiary care), training communities and health care workers in emergency preparedness and response, engaging communities in building resilience, and presenting innovative strategies to improve accessibility to aspects of PHC with limited resources (e.g., palliative care), in various contexts.

## Conclusion

Globally, it is recognised that young people have the responsibility for continuously advocating to improve PHC at global, regional and local levels in order to influence change within their communities from a grassroots level. As an innovative CoP with demonstrated benefits to individuals, communities and the global youth movement, the YLN offers a powerful example of how youth can be meaningfully engaged in strengthening PHC.

The CoP model’s *strengths* are that it is dynamic, flexible and driven by passionate young PHC advocates, particularly valuable when facing PHC challenges such as the COVID-19 crisis. CoP members can continue to work tirelessly in both individual and collaborative capacities to (a) increase PHC’s visibility and (b) bring PHC closer to their communities.

Potential *weaknesses* or challenges are in ensuring sustainability within a changing context and limited facilitation support. However, determination, shared identity, agility and commitment allow CoP members to strive together to achieve a better future for PHC. Another challenge of the YLN is that of continuous online engagement with the entire CoP. The time difference between continents contributes to limiting group engagement. However, with a clear plan and group facilitator, this challenge can be overcome.

Moving forward, the YLN seeks *opportunities* to continue meaningful engagement with other active youth groups and experts in the field working towards the SDGs, to form a broader base and find shared, innovative solutions to PHC challenges. Collaboration and engagement with other active youth groups might contribute towards sustaining the YLN CoP.

PHC has never been more important than in the current climate of the COVID-19 pandemic. While this *threatens* the focus for young leaders, it also presents a chance to advocate strongly in a time where the whole world is talking about health. For the YLN, there is a desire to use the foundation of the CoP to share knowledge, evidence and resources to inform clinical and public health activities, policy initiatives, health activism and research projects that would serve to advance the science and practice of PHC and improve health outcomes for communities globally.
